# Identification of Scleractinian Coral Recruits Using Fluorescent Censusing and DNA Barcoding Techniques

**DOI:** 10.1371/journal.pone.0107366

**Published:** 2014-09-11

**Authors:** Chia-Min Hsu, Stéphane de Palmas, Chao-Yang Kuo, Vianney Denis, Chaolun Allen Chen

**Affiliations:** 1 Biodiversity Research Center, Academia Sinica, Nangang, Taipei, Taiwan; 2 Institute of Oceanography, National Taiwan University, Taipei, Taiwan; 3 ARC Centre of Excellence for Coral Reef Studies, James Cook University, Townsville, Australia; 4 Taiwan International Graduate Program (TIGP)-Biodiversity, Academia Sinica, Nangang, Taipei, Taiwan; University of Connecticut, United States of America

## Abstract

The identification of coral recruits has been problematic due to a lack of definitive morphological characters being available for higher taxonomic resolution. In this study, we tested whether fluorescent detection of coral recruits used in combinations of different DNA-barcoding markers (cytochrome oxidase I gene [COI], open reading frame [ORF], and nuclear *Pax*-*C* intron [PaxC]) could be useful for increasing the resolution of coral spat identification in ecological studies. One hundred and fifty settlement plates were emplaced at nine sites on the fringing reefs of Kenting National Park in southern Taiwan between April 2011 and September 2012. A total of 248 living coral spats and juveniles (with basal areas ranging from 0.21 to 134.57 mm^2^) were detected on the plates with the aid of fluorescent light and collected for molecular analyses. Using the COI DNA barcoding technique, 90.3% (224/248) of coral spats were successfully identified into six genera, including *Acropora*, *Isopora, Montipora*, *Pocillopora*, *Porites*, and *Pavona.* PaxC further separated *I*. *cuneata* and *I*. *palifera* of *Isopora* from *Acropora*, and ORF successfully identified the species of *Pocillopora* (except *P*. *meandrina* and *P*. *eydouxi*). Moreover, other cnidarian species such as actinarians, zoanthids, and *Millepora* species were visually found using fluorescence and identified by COI DNA barcoding. This combination of existing approaches greatly improved the taxonomic resolution of early coral life stages, which to date has been mainly limited to the family level based on skeletal identification. Overall, this study suggests important improvements for the identification of coral recruits in ecological studies.

## Introduction

Coral recruitment, defined as the number of coral larvae entering the adult population, plays a critical role in the resilience of coral populations [1], [2]. Understanding the diversity of coral recruits, their spatial and temporal dynamics, and the processes maintaining local coral assemblages are particularly important because coral reefs worldwide are declining as a consequence of natural and anthropogenic disturbances [3], [4].

Artificial substrates (*e.g*., settlement plates) have been widely used to study scleractinian coral recruitment in the past three decades [5]–[7]. Coral larvae settle on plates and secrete a calcareous skeleton within a few hours after settlement [8]. Skeletal structures remain as a record after the polyp dies unless they are overgrown by other benthos or are removed by grazers or other physical mechanisms (*e.g*., strong waves) [8]. Therefore, this technique has been used despite post-settlement mortality to examine spatio-temporal variations in recruitment patterns [9], [10], dispersal of scleractinian corals [11], effects of competition on coral recruits [5], regional variation in recruitment [12], processes that maintain coral populations [13], and degradation of reefs [14], [15].

However, juvenile corals have only a few useful taxonomic characters [16], making their identification particularly difficult. Whereas low scleractinian coral diversity allows identification to genus or species levels in the Caribbean [17], it is at best limited to generic identification in the Indo-Pacific where coral assemblages are much more diversified. After raising juveniles of over 30 common coral species representing 21 genera from 15 scleractinian families, Babcock [18] and Babcock et al. [16] concluded that only three families (Acroporidae, Pocilloporidae, and Poritidae) could consistently and successfully be identified but that even greater taxonomic resolution could be achieved by using corallite ultrastructure. Consequently, the remaining unidentified coral juveniles are usually recorded as “others” in most published studies (see Table 1). The lack of higher taxonomic resolution of coral recruits has retarded their contribution toward understanding the dynamic processes of coral populations and coral communities in which operational taxonomic units (OTUs) should normally be at least to the genus level [19]–[21].

**Table 1:** Lowest levels of coral recruit taxonomic identification in the literature.

Spat ID resolution	Other (%)	Material	Recruitment rate	Study site	Reference
Family/other	8.9–13.1	Ceramic plate	15.6–81.1 spats plate^−1^	Australia	[68]
Family/other	2.4–5.1	Clay tile	1.25–219 spats plate^−1^	Australia	[54]
Family/other	1.7–5	*Terra cotta* tile	0.2–1.7 spats 100 cm^−2^	Australia	[69]
Family/other	<1	Ceramic tile/PVC plate/carbonic plate	0–75 spats plate^−1^	Taiwan	[56]
Family/unidentified	16.3	*Terra cotta* tile	285 to 772 spats m^−2^ yr^−1^	Indonesia	[14]
Family/other	16.1	Ceramic plate	2.53 spats plate^−1^	Eilat	[9]
Genus/unidentified	25	Artificial slate plate	2 spats m^−2^	Japan	[70]
Species/species group	1.4–2.8*	Ceramic plate	<0.1–54.8 spats plate^−1^	Caribbean	[19]
Family/genus/species	0.3*	Ceramic plate	0.54–2.96 spats m^−2^ yr^−1^	Florida	[39]
Family	35.1	Natural lime stone plate	20.2 spats plate^−1^	Indonesia	[25]
Family/genus/species	< 5	PVC plate	32.5 spats m^−2^	Taiwan	[71]
Family/other	0–17	Acrylic plate	7.2 spats m^−2^ yr^−1^	Thailand	[15]
Family/other	49	Concrete/*terra cotta* plate/cleared reef substrate	0–8.5 spats plate^−1^; 1.42–1.72 spats 100 cm^−2^	Indonesia	[72]
Family/other	55–85	*Terra cotta* tile	113–909 spats m^−2^	Australia	[73]
Family/other	< 10	*Terra cotta* tile	283–1043.8 spats m^−2^	Taiwan	[58]
Family/other	< 10	Ceramic tile	8.0–116.4 spats m^−2^	Taiwan	[57]
Family/genus/species	9.7*	*Terra cotta* tile	0.38–9 spats plate^−1^; 21.3–553.5 m^−2^	Taiwan	This study

*: Application with molecular analysis.

Recently, fluorescent censusing has become a helpful field tool for the early detection of coral recruits [22]–[25], as small corals living under cryptic structures like macroalgae or turf or within crevices are easily detected in comparison with white light observations. This technique has significantly increased our capacity to detect coral recruits, but their identification remains problematic as only a few can be identified during a limited period of time after settlement [16].

DNA barcoding is a taxonomic method that uses a short genetic marker in an organism's DNA to identify it as belonging to a particular species [27]. A 658-bp region of the mitochondrial (mt) cytochrome *c* oxidase subunit I (COI) gene has been widely used in diverse applications of DNA barcoding in animals [28], [29]. However, the slow rate of evolution in mitochondrial genomes has called into question the usefulness of COI DNA barcoding for identifying coral species (and anthozoans in general) [19], [30]–[36]. Nevertheless, phylogenetic analyses of COI across a wide range of scleractinian species shows that it is consistent with coral taxonomy to the genus level [37], [38], and even to the species level in *Stylophora* [32], suggesting that COI can serve as a genetic tool for broadening the taxonomic resolution of corals. However, most of those studies were with adult corals and only a few attempts have been done on coral recruits. Shearer et al. [19] first applied genetic marker COI and species-specific RFLP patterns to identify some of the coral recruits sampled at two areas in the Caribbean. Rubin et al. [39] later used COI and cytochrome *b* gene to increase the taxonomic resolution of coral recruits in damaged reef sites in southeast Florida. In the Pacific region, only Suzuki et al. [21] have identified the dominant *Acropora* species recruits in the Ryukyu Archipelago using two-step molecular sorting with mitochondrial and nuclear markers.

Here, we describe diversity in coral recruits collected from plates deposited during April 2011 to September 2012 at different sites around Kenting National Park, Taiwan. We combined fluorescent censusing and DNA barcoding techniques to determine if those tools can improve the resolution of coral recruit identification. Finally, we recommend a combination of molecular markers to use in future studies to significantly increase our knowledge of critical ecological processes like coral recruitment.

## Materials and Methods

### Ethics statement

This study was conducted with permission of the Kenting National Park authorities, Taiwan. Samples were collected as part of long-term ecological monitoring research with permit nos. 1002901146 and 1010001032 for the years 2011 and 2012, respectively. All collecting procedures were done with proper precautions for minimizing impacts to reefs.

### Study area, settlement plate deposition, and plate retrieval

**Figure 1:**
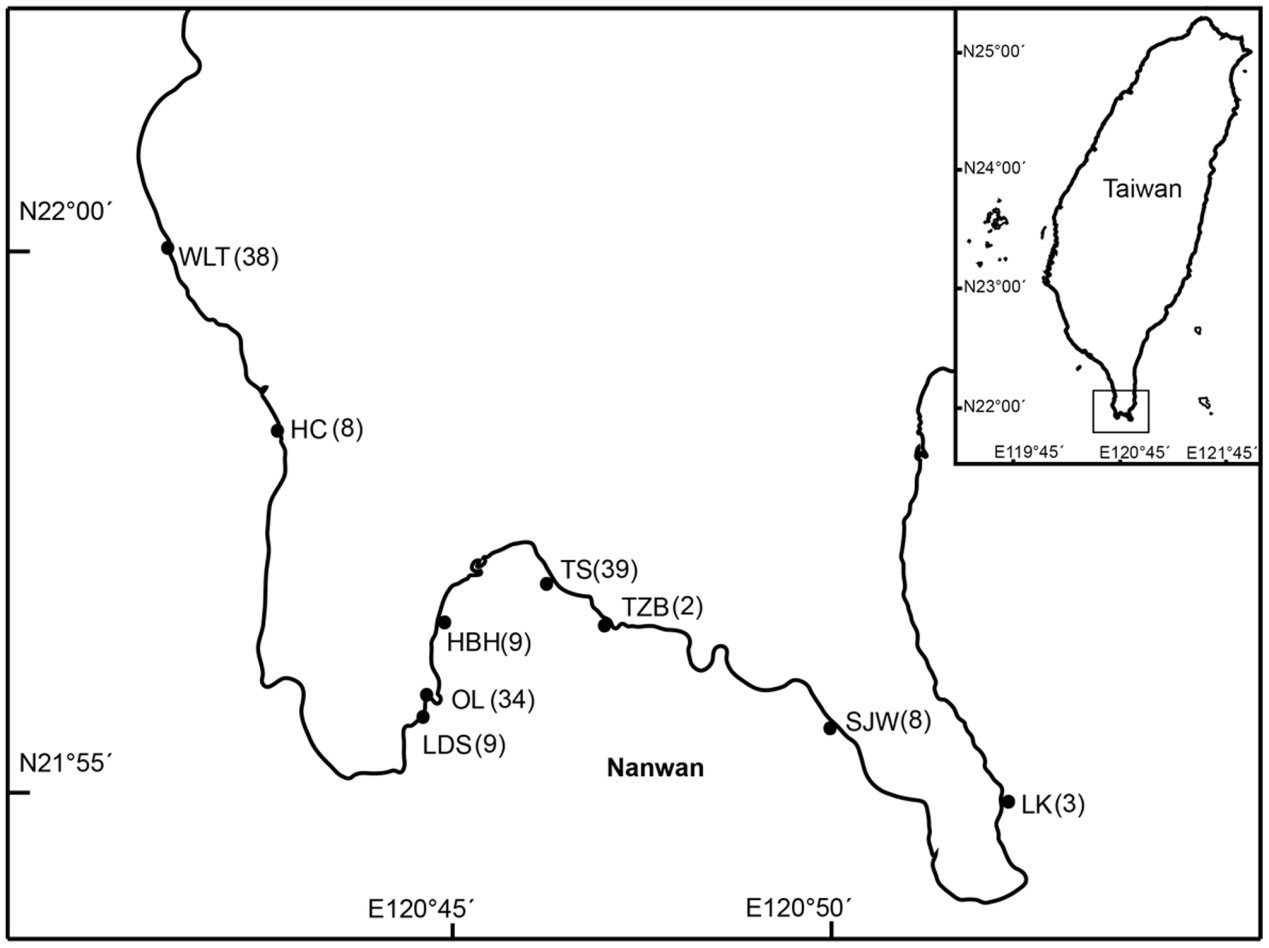
Map of study sites in southern Taiwan. The number of retrieved plates is indicated in parentheses. Abbreviations of sites: WLT, Wanlitung; HC, Hungchai; LDS, Leidashih; OL, outlet of warm water discharge from nuclear power plant; HBH, Hobihu; TS, Tiaoshi; TZB, Tantzei Bay; SJW, Shinjaowan; LK, Longkeng.

This study was conducted along the coast of Kenting National Park (KNP), Taiwan (Fig.1). Fringing reefs go to depths of 30 m, but most corals thrive at depths <10 m. Nine long-term monitoring sites established by KNP were selected: Wanlitung (WLT), Hungchai (HC), Leidashih (LDS), warm water discharge outlet from a nuclear power plant (OL), Hobihu (HBH), Tiaoshi (TS), Tantzei Bay (TZB), Shinjaowan (SJW), and Longkeng (LK) (Fig. 1). In 2010–2011, total coral coverage ranged between 67.0% at OL and 2.9% at HC [40], [41] (see details in Table 2). Hard corals contributed to a maximum of 67.0% of the benthic community at OL and to a minimum of 2.8% at HC. Soft corals were absent at OL, but covered up to 37.3% of the substrate at LDS. Sedimentation and nutrient discharge negatively affected coral coverage at the different sites and are known to be the main factors in reef degradation at Kenting [42]. Reefs further suffer from overfishing and habitat destruction as well as natural disturbances such as typhoons, causing a significant reduction in living coral coverage at many sites around KNP [43]–[45]. Overall, our study focuses on sites encompassing a range of environmental conditions presenting different levels of degradation.

**Table 2:** Recruitment rate and coral coverage among study sites.

Site	LDS	TZB^c^	SJW	TS	HC	HBH	LK	WLT	OL
GPS	N21°55.807′	N21°56.998′	N21°55.413′	N21°57.163′	N21°58.352′	N21°56.583′	N21°54.465′	N21°59.701′	N21°55.888′
	E120°44.689′	E120°46.554′	E120°49.760′	E120°46.203′	E120°42.910′	E120°45.119′	E120°51.676′	E120°42.216′	E120°44.694′
Short-term recruit (no. plate^−1^, n = 3)									
4 mo.	7.0	5 (n = 2)	2	0.6	1.6	4	-	2.3	0
5.5 mo.	14.6	-	1	1.3	0.6	0	-	1	-
7 mo.	5.3	-	5 (n = 2)	5	2 (n = 2)	0	-	1.6	1
Long-term recruit (no. plate^−1^, n = 10)									
4 mo.	-	-	-	0.9	-	-	-	0.3	0
9.5 mo.	-	-	-	2.7	-	-	-	1.5	0.8 (n = 9)
16.5 mo.	-	-	-	1.4	-	-	1.3 (n = 3)	0.6 (n = 9)	0.2 (n = 9)
Recruit (no. plate^-1^)^a^	9	5	2.7	1.8	1.3	1.3	1.3	1.0	0.4
Recruit (no. m^-2^)^b^	553.7	307.6	169.2	111.9	84.2	81.8	81.8	62.6	23.3
Adult hard coral (%)	23.7	16.3	20.5	9.2	2.8	52.1	26.5	16.6	67.0
Soft coral (%)	37.7	0.1	0.4	0.6	0.1	7.7	4.1	0.3	0.0
Dominant genus	*Sinularia*	*Montipora*	*Montipora*	*Montipora*	*Favia*	*Acropora*	*Heliopora*	*Porites*	*Montipora*
(%)	35.9	7.2	46.9	37.0	14.5	39.5	35.0	45.9	21.7
	*Sarcophyton*	*Favites*	*Favites*	*Favites*	*Heliopora*	*Porites*	*Pocillopora*	*Heliopora*	*Galaxea*
	15.2	1.4	8.5	12.2	13.6	14.2	12.0	10.9	21.2
	*Favia*	*Favia*	*Porites*	*Porites*	*Porites*	*Montipora*	*Sinularia*	*Astreopora*	*Favites*
	8.5	1.2	6.1	8.2	10.9	9.4	10.4	4.8	5.4

Adult coverage survey in 2010-2011 and recruit survey in 2011–2012. Abbreviations of sites: WLT, Wanlitung; HC, Hungchai; LDS, Leidashih; OL, outlet of warm water discharge from a nuclear power plant; HBH, Hobihu; TS, Tiaoshi; TZB, Tantzei Bay; SJW, Shinjaowan; and LK, Longkeng.

a overall recruitment rate, number of spats per plate

b overall recruitment rate, number of spats per square meter

c adult survey is ∼50 m away from plates

Terra cotta plates (12.5×13×1 cm) were deposited at every site, fastened to 10-cm stainless steel expansion bolts imbedded into natural substrates at ∼5 m depths with a pneumatic drill, with bolts separated by distances of 20–50 cm. Plates were secured in horizontal positions with two stainless steel washers and a hex nut about 3–5 cm above the substrate that was available for coral recruitment in close proximity to adult colonies. Plates with different deposition/collection dates as well as time spent underwater were pooled for this study to maximize the diversity of coral recruits by integrating the wide variety of pre- and post-settlement processes at the different study sites. Settlement plates were deployed for short-term (4 to 7 months) and long-term (4 to 16.5 months) surveys. The first deployment of plates was conducted just before the coral spawning season, which extends from May to September in southern Taiwan (some brooder species can reproduce year round) [46]. Therefore, for the short-term survey, three plates were deployed in April 2011. Plates were retrieved and replaced in August 2011 and February and September 2012. For the long-term survey, thirty additional plates were set out at WLT, OL, and TS in April 2011. Ten randomly selected plates were sampled in August 2011, February 2012, and September 2012. TZB was sampled only once during the short-term survey in August 2011. At LK, only three plates were collected after 16.5 months because of logistics difficulties and weather conditions. In total, 159 plates were deposited in the field for this study (see details in Tables 2, 3).

**Table 3:** Detailed information regarding the number of plates deposited/retrieved, number of spats collected/identified, number of identified spats from different genera, and recruitment rate/identification rate per plate at all sites.

Site	LDS	TZB	SJW	TS	HC	HBH	LK	WLT	OL	Sum
Plate deposited	9	3	9	39	9	9	3	39	39	159
Plate retrieved	9	2	8	39	8	9	3	38	34	150
Spat collected	81	10	22	71	11	12	4	39	13	263
Spat identified	71	10	21	53	10	9	3	37	10	224
Spat unidentified	10	-	-	7	1	3	-	1	2	24
Spat misidentified	-	-	1	4	-	-	-	-	1	6
Other cnidarians										
Milleporian	-	-	-	1	-	-	-	1	-	2
Zoanthid	-	-	-	3	-	-	1	-	-	4
Actiniarian	-	-	-	3	-	-	-	-	-	3
Recruitment rate^a^	9.0	5.0	2.8	1.8	1.4	1.3	1.3	1.0	0.4	1.8
Identification rate^b^	87.6	100	100	88.3	90.9	75	100	97.4	83.3	90.3
Identified by COI										
*Montipora*	-	-	-	-	-	-	-	1	-	1
*Pavona*	-	-	-	2	-	-	-	1	-	3
*Isopora*	-	7	-	-	-	-	-	-	-	7
*Acropora*	-	1	4	6	-	-	-	-	-	11
*Porites*	6	-	1	9	-	-	1	-	2	19
*Pocillopora*	65	2	16	36	10	9	2	35	8	183
Identified by ORF										
Type α	3	-	-	-	-	-	-	-	-	3
Type β	7	-	-	1	-	-	-	1	1	10
Type γ	3	-	3	10	-	-	-	7	-	23
Type δ	-	-	-	-	-	-	-	-	-	0
Type ε	15	-	-	-	-	-	1	-	-	16
*P. eydouxi/meandrina*	2	-	1	2	1	-	-	5	-	11
Spat ORF typed	30	-	4	13	1	-	1	13	1	63
Spat not typed	35	2	12	23	9	9	1	22	7	120

Spat number for different genera is listed. The order of sites was based on the recruitment rate per plate from the highest to lowest. Abbreviations of sites are as described in Table 2.

a number of spat collected per retrieved plate

b number of coral spat identified from collected spats (*e.g*., total 263: misidentified 6, other cnidarians 9  =  248, identified spat 224, collected spat 248  =  0.903)

Plates collected were placed individually in Ziploc bags underwater, transported to the laboratory, and processed as soon as possible to detect and sample coral recruits.

### Coral spat identification, collection, and preservation

Coral spats were defined as individuals that successfully settled and survived by the time of sampling. This corresponds to effective recruitment; *i.e.,* spats surviving post-settlement mortality and having a chance to join the population. Live spats were spotted under excited blue light using long-pass (wavelength >500 nm) barrier filter glasses (Night Sea VG1) in a darkened room. Fluorescent and normal images of coral spats were photographed under blue and white light for confirmation of coral spats or other fluorescing organisms using a digital Canon Kiss 4 camera with a 100 mm macro lens. Photographic lighting was provided by a dive light (Night Sea BW-1) by toggling between white light without a lens filter and blue light excitation with a long-pass lens filter (Night Sea BB67) mounted on the lens. Spats were then removed from plates and fixed separately in 1.5 mL Eppendorf tubes containing 100% ethanol for further molecular analysis. The above workflow was processed within 6–12 hours after plates were collected from the sea on each retrieval day. The time spent by two people inspecting each plate was 30 min on average (10–120 min) depending on how many recruits were found.

### Genomic DNA extraction, polymerase chain reaction, and DNA sequencing

DNA extraction was conducted using a modified high-salt method [47]. After removing the ethanol, individual coral spats were lysed with 10 µL of 10 mg/mL proteinase E and 200 µL lysis buffer (0.25 M Tris, 0.05 M EDTA at pH 8.0, 2% sodium dodecylsulfate (SDS), and 0.1 M NaCl) overnight at 55°C in a dry bath. Equal volumes of 210 µL of 7 M NaCl were added to the lysed slurry and gently inverted. The mixture was then transferred to a DNA spin column (Viogene) sitting in a 2 mL collection tube and centrifuged at 8000 rpm for 1 min. The column was washed twice with −20°C chilled 70% ethanol by centrifuging at 8000 rpm for 1 min at each step, with an additional centrifugation step at 8000 rpm for 3 min to dry the spin column. The column was dried at 37°C for 15 min and DNA eluted according to spat size in 20 ∼ 50 µL ddH_2_O, with a final centrifugation at 13,000 rpm for 3 min. PCR amplification was performed in a 50 µl total volume with a final concentration of 1X Buffer (200 nM Tris-HCl, pH  =  8.4, 500 mM KCl), 0.1 mM dNTP, 0.1 mM of each primer, 3 mM MgCl_2_, and 0.5 U Taq DNA Polymerase (Invitrogen). The COI primers used were described by Folmer et al. [48] as LCO1490: 5′-GGT CAA CAA ATC ATA AAG ATA TTG G-3′ and HCO2198: 5′-TAA ACT TCA GGG TGA CCA AAA AAT CA-3′. Thermal cycling protocol conditions consisted of denaturing by heating at 95°C for 3 min followed by 30 cycles at 95°C for 60 s, 45°C for 60 s, and 72°C for 90 s. Amplified products were visualized using 2% agar gel electrophoresis and nucleotide sequences determined with an ABI 3730 Genetic Analyzer. Other sequences were acquired from the scleractinian DNA barcoding database using BOLD v2.5 [49]. Coral recruits identified by COI as *Acropora/Isopora* species were further subjected to a second PCR reaction with the *Pax-C* intron [50]. The primers were *PaxC*_intron-FP1: 5′-TCC AGA GCA GTT AGA GAT GCT GG-3′ and *PaxC*_intron-RP1: 5′-GGC GAT TTG AGA ACC AAA CCT GTA-3′. Coral recruits identified by COI as *Pocillopora* were subsequently examined by PCR using the ORF region [51]. ORF primers were FATP6.1: 5′-TTT GGG SAT TCG TTT AGC AG-3′ and RORF: 5′-SCC AAT ATG TTA AAC ASC ATG TCA-3′. PCR amplifications and DNA sequencing of PaxC and ORF regions were performed as described above.

### Phylogenetic analysis

DNA sequences were assembled using SeqMan (DNASTAR, USA) and then sequence alignments and analyses were conducted using MEGA v5.2 [52]. The COI sequences of 216 scleractinian species obtained from the BOLD database (www.barcodeoflife.org) were retrieved and compared to the 224 sequences (NCBI, GenBank accession numbers KM214845- KM215068) from this study. Because size differences in the scleractinian DNA COI database introduced a significant number of gaps, short or incomplete sequences were removed from the analysis in order to use only sequences with >500 bp. Sequences were aligned using CLUSTAL W with default settings. After a model test, neighbor-joining (NJ) cluster analysis was conducted with 1000 bootstrap replications using MEGA 5.2 [52]. After genus sorting based on cluster analysis, PaxC sequences of *Acropora*/*Isopora* samples (NCBI, GenBank accession numbers KM214830- KM214844) were aligned and trimmed to a length of 335 bp to compare with known *Isopora* sequences from adult colonies and *Acropora* sequences downloaded from NCBI. The sequences of ORF from *Pocillopora* spats (NCBI, GenBank accession numbers KM215069- KM215131) were aligned with sequences from [53], with a final alignment of 800 bp. The same phylogenetic analyses were performed as described above.

## Results

### Fluorescent detection of coral spats

**Figure 2:**
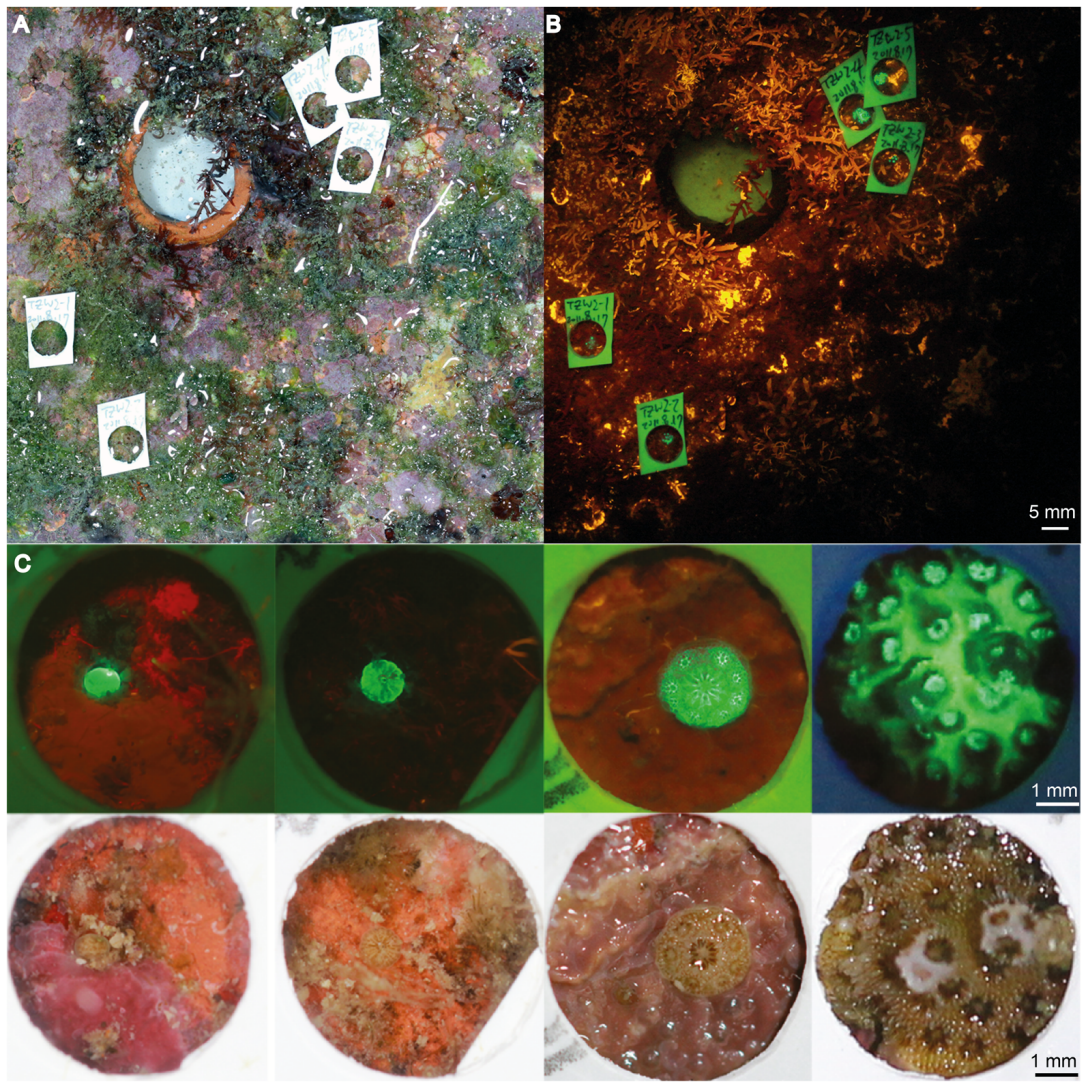
Coral spat settled on recruitment plates. (A) Normal image of coral spats on plate; (B) fluorescent image of coral spats on plate; and (C) different early life stages of settled *Pocillopora* corals.

A total of 150 out of 159 plates were successfully retrieved from the nine sites between August 2011 and September 2012 (Table 3). In total, 263 coral-like spats were found and collected with the aid of fluorescent lighting and observed under white light (Fig. 2A). Sediment, turf algae, and macroalgae generally covered the top surfaces of the plates; therefore, the majority of coral spats settled on the bottom surface and/or the vertical edges of plates. Other benthic organisms, such as ascidians, bryozoans, barnacles, and sponges could also be commonly found on the plates.

Under blue light excitation, most of the coral spats emitted a green fluorescence against a contrasting red or orange chlorophyll fluorescence from algae (Fig. 2B). Ascidians and bryozoans that emitted green fluorescence were avoided as much as possible in subsequent sampling. A few spats, however, emitted red fluorescence (data not shown) and were further spotted with the aid of white light.

**Figure 3:**
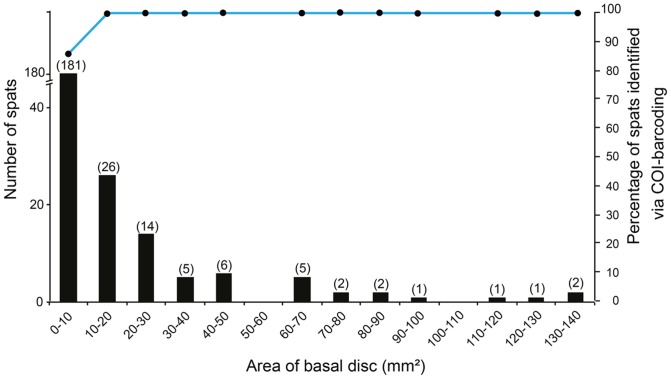
Efficiency of COI barcoding on spats of different sizes. All sizes found in this study were successfully identified with COI barcoding, except those <10 mm^2^, which had an 86.7% (157/181) identification rate. The bar chart represents the number of spats and the dot with blue line represents the identification rates for different sizes.

The smallest spats were particularly difficult to spot with the use of white light only, but by combining this approach with fluorescent lighting we were able to efficiently detect live spats ranging between <1 mm and 6 mm in diameter (Fig. 2C). Most of the spats (72.9%, 181/248) were small, with basal areas ranging from 0.21 to 9.95 mm^2^ (with respective maximum diameters of 0.52 mm and 4.26 mm, Fig. 3).

### DNA barcoding of coral spats

Among the 263 coral-like spats collected, nine were identified visually as other cnidarians and six as other organisms (mainly algae, ascidians, and polychaetes; see ‘Other cnidarians’ and ‘misidentified’ in Table 3). The remaining 224 of 248 were successfully phylotyped with COI DNA sequences at an overall success rate of 90.3% (Table 3). The basal area of the smallest spat identified in this study with COI was 0.36 mm^2^. The smallest spats (basal area <10 mm^2^) appear to be the most difficult to phylotype, with only 86.7% (157/181) of them being successfully identified. All larger spats were successfully phylotyped (Fig. 3).

**Figure 4:**
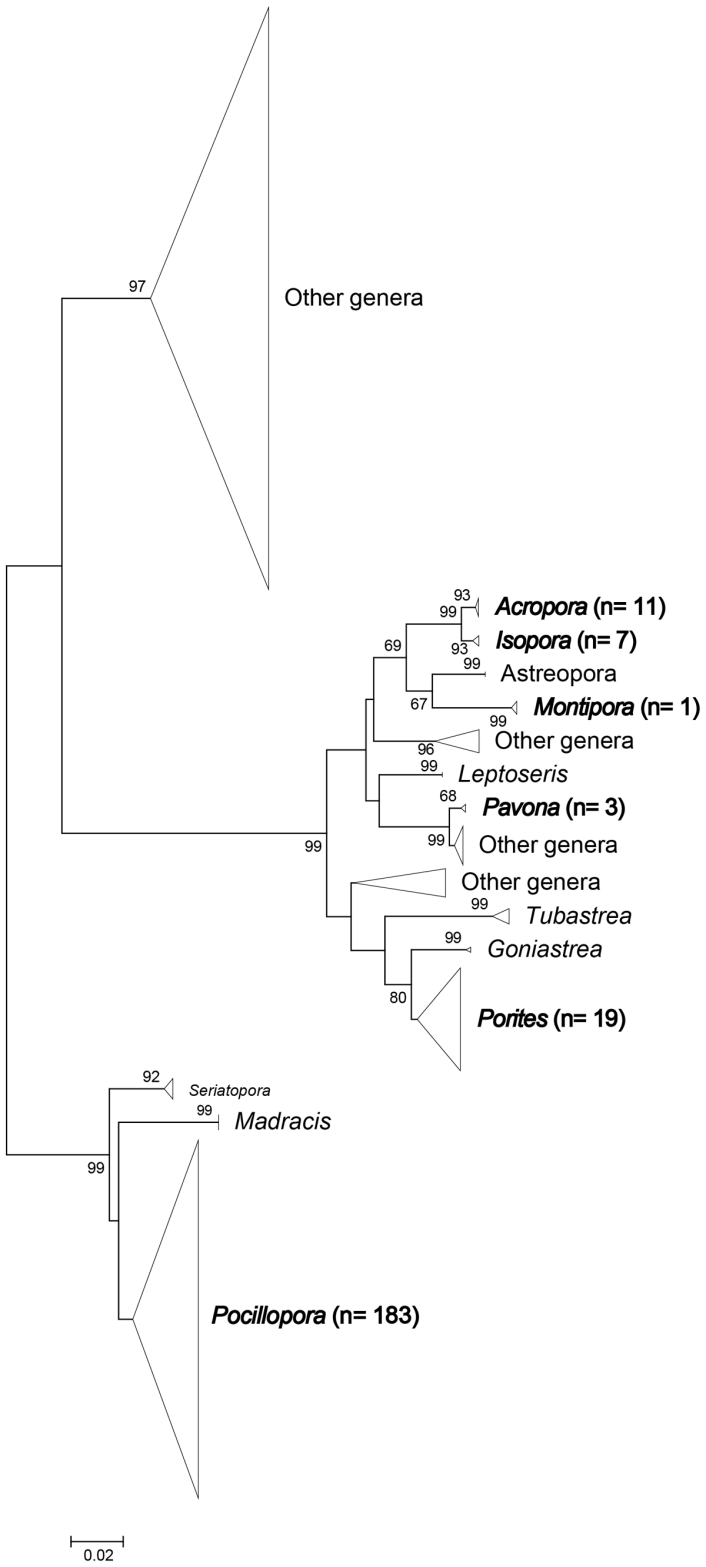
NJ tree of scleractinian COI sequences. Neighbor-joining tree of mitochondrial COI sequence constructed by Tamura three-parameter model with 1000 bootstrap replications. The genus with its sample number in parentheses labeled in bold was discovered from a settlement plate in this study. All other generic sequences were obtained from the database of the Barcode of Life (www.barcodeoflife.org).

Six genera (*Acropora, Isopora, Montipora, Porites, Pavona,* and *Pocillopora*) were identified based on COI phylogenetic analysis (Fig. 4). *Pocillopora* (n  =  183) represented 81.7% of identified coral spats, with *Porites* (8.5%, n  =  19) and *Acropora* (4.9%, n  =  11) as the second- and third-most abundant groups in our samples (Table 3). *Pavona* (n  =  3) was detected in TS and WLT, whereas *Isopora* (n  =  7) and *Montipora* (n  =  1) were found only in TZB and WLT, respectively (Table 3).

**Figure 5:**
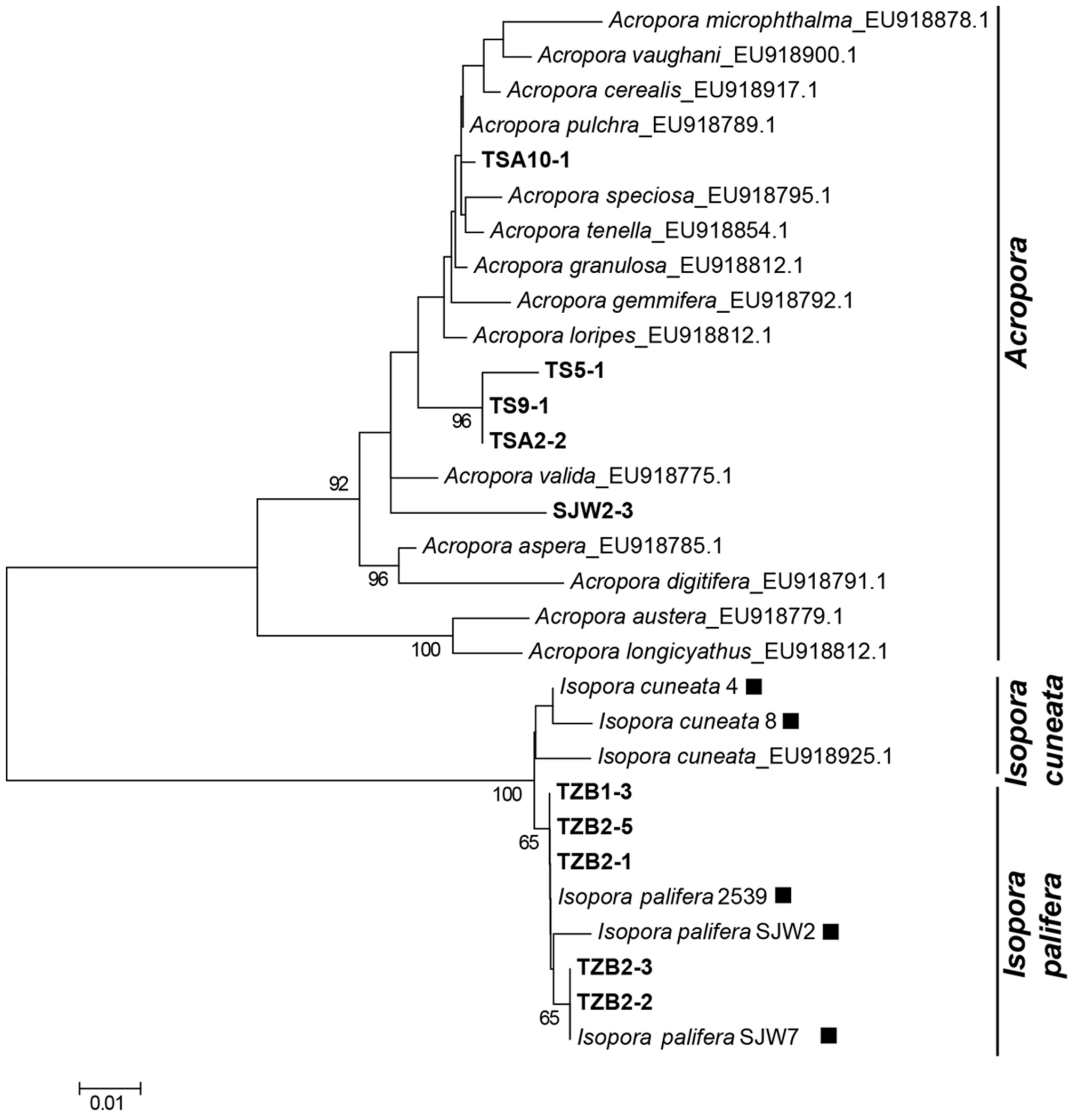
NJ tree of PaxC sequences for *Acropora*/*Isopora* identification. (A) Neighbor-joining tree of PaxC constructed by Tamura three-parameter model with 1000 bootstrap replications. The sequences of coral spat and reference corals in this study are labeled in bold and filled squares, respectively. All other PaxC sequences are from reference [67].

For the Acroporidae, PaxC also clearly separated *Acropora* from *Isopora* (Fig. 5) and allowed us to distinguish *I. palifera* from *I. cuneata* (supported by 100% bootstrap in the NJ tree, Fig. 5).

**Figure 6:**
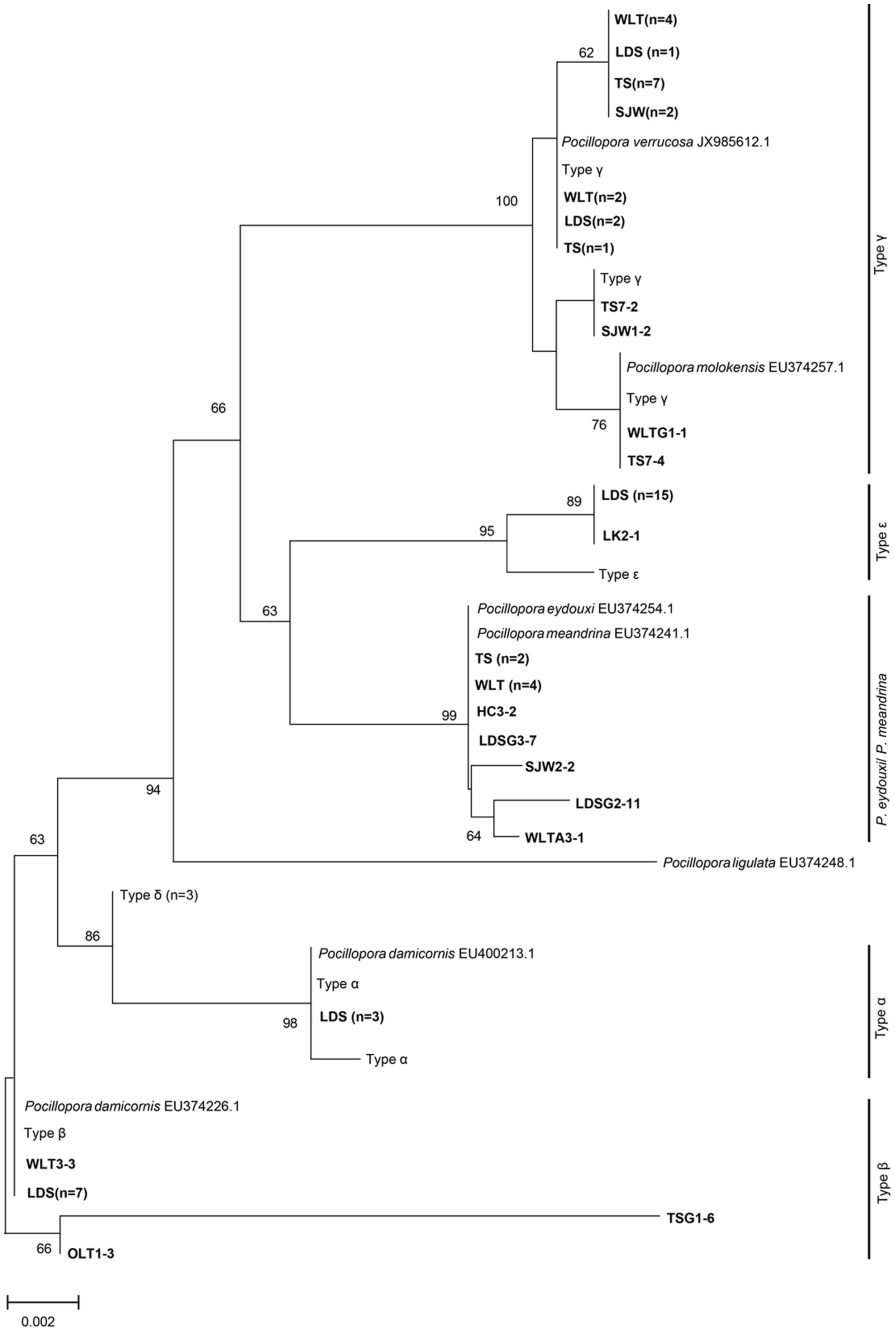
NJ tree of the mitochondrial ORF sequence of *Pocillopora*. Neighbor-joining tree of ORF sequence constructed by Tamura 92 model with sequences from [51] and [53] (personal communication). Samples from different sites and numbers in parentheses labeled in bold were discovered from the settlement plate in this study. There are four mitochondrial haplotypes (alpha, beta, gamma, and epsilon) found in *P. damicornis*.

ORF sequences produced higher taxonomic resolution in the genus *Pocillopora* (Fig. 6). Among the 183 *Pocillopora* spats, a subsample of 63 recruits were amplified and sequenced (Table 3). Six *Pocillopora* ORF sequences were successfully identified as *Pocillopora* α, β, γ, δ, ε and *P. eydouxi/P. meandrina,* following Schmidt-Roach et al. [53].

### Recruitment among sites

**Figure 7:**
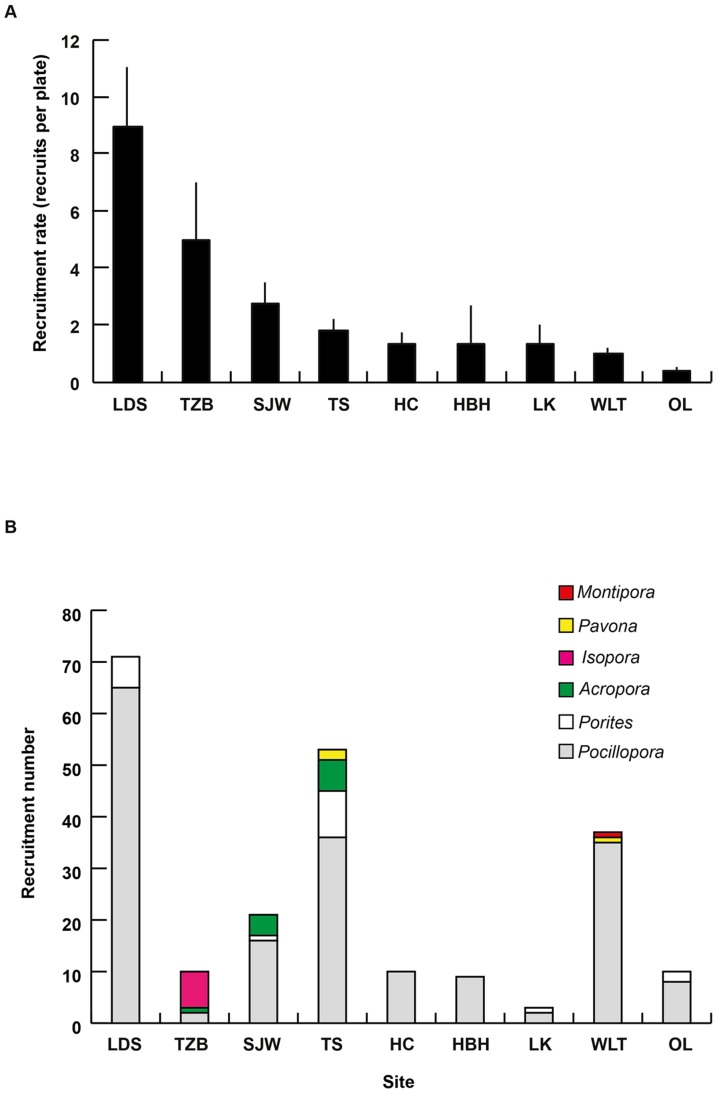
Recruitment among study sites. (A) Site recruitment rate ranking (from the highest to lowest). Error bars represent the standard error (SE). (B) Recruitment composition was identified by COI sequences among sites.

The total number of recruits observed alive on the plates ranged from 81 at LDS to 4 at LK (Table 3), with major differences in the number of plates between sites, date of deposition/collection, and time spent underwater making it difficult to compare among sites. However, some obvious trends in the spatial patterns of recruitment were observable when data were reported by the number of plates examined (Fig. 7A). For all plates combined, the mean density of coral recruits was 1.8 ± 0.2 (mean ± SE) per plate. LDS had the highest recruit density at 9.0 ± 2.0 (mean ± SE), while OL values were almost 23x lower at a density of 0.4 ± 0.1 (mean ± SE) (Tables 2, 3).

*Pocillopora* spats were identified at every site, whereas *Isopora* was only found at TZB and *Montipora* only at WLT (Table 3, Fig. 7B). In HC and HBH, only *Pocillopora* was successfully phylotyped. In contrast, TS had the highest generic diversity of coral spats, with *Acropora, Porites, Pavona,* and *Pocillopora* spats being successfully detected using COI.

## Discussion

Most studies to date have been limited to identifying coral recruits to the family level. They usually examined skeletal characteristics by microscope, often disregarding post-settlement mortality when estimating recruitment. By combining different tools like fluorescence censusing and DNA barcoding markers, this report offers an original approach for improving the analysis of effective recruitment by quantifying the number of survivors actually added to the population. Overall, this study suggests important improvements for identifying coral recruits in ecological studies.

### Detection of coral recruits using fluorescence censusing

Fluorescence censusing has significantly improved our ability to detect coral recruits [23]. In the field, it has even been used during the daytime to detect small sized or cryptic juvenile corals and in the early detection of coral spats [22], [24]–[26]. Whereas recruits detected with white light present an average diameter usually >5 mm [22], [24], the fluorescence census technique produces excellent resolution for sizes down to 1 mm in diameter [24], [26]. We demonstrate that, under good laboratory conditions, we were able to detect recruits with diameters as low as 0.52 mm (basal area 0.21 mm^2^), as observed in [22], [25]. Detections in the field are usually limited by the distance between the camera and the substrate [22], [24]–[25] as well as the intensity of fluorescent light underwater. In 2006, Schmidt-Roach et al. [25] examined coral recruitment on Meras Reef, Manado, Indonesia. Using limestone plates left under water for four months, they recorded a total of 808 recruits via microscopic examination after treatment with a chloride solution. Only 28.6% (231) of them had been previously spotted in the field with a fluorescence census. Aside from dead recruits, the authors suggested that low fluorescence signals from small recruits could explain this difference (see also [22]). In our study, however, we were able to detect even the smallest recruits (0.52 mm diameter) with fluorescence censusing under laboratory conditions in a dark room. Fluorescence also facilitated the detection of tiny spats (Fig. 3) that were hidden beneath turf or macroalgae. However, we recommend its use in combination with white light because some spats (especially *Porites*) exhibit very low fluorescence. Additionally, the use of white light may avoid sampling other organisms presenting highly similar fluorescence levels, including other cnidarians, polychaetes, ascidians, and some algae.

### DNA barcoding of coral recruits revealed higher resolution at different taxonomic levels

In the last three decades, the analysis of the coral recruit diversity has relied mainly upon the microscopic examination of skeletal characters after soft tissues have been removed [18]. Even with scanning electron microscope photographs [16], the taxonomic resolution of coral recruits was previously limited essentially to the family level, mainly Acroporidae, Poritidae, and Pocilloporidae. Unidentified coral spats were categorized as “others” in most studies (Table 1), and the percentage of unidentified coral recruits based on these four taxa could be as high as 85% (Table 1). In extreme cases, such as in the first 24 h after settlement, all recruits have even been classified as unknown [26]. Therefore, studies on early life stages to date have contributed little to our knowledge of the role of coral recruitment in explaining coral community structure, as a minimum resolution to the genus level is usually required [54], [55]. Consequently, higher taxonomic resolution is needed to document this fundamental ecological process for an adequate understanding of coral community resilience.

While some studies show limitations to the use of DNA barcoding techniques on corals, they provide useful tools for documenting diversity at least to the generic level. However, and surprisingly, only a few studies to date have tried to use these techniques to identify coral recruits [19], [21], [39]. We document herein that the use of a combination of three selected molecular markers (COI, PaxC, and ORF) can significantly improve the taxonomic resolution of recruitment studies in comparison with previous studies conducted solely by microscopic inspection of recruit skeletons (see Table 1), and are even useful in the identification of some species. During a 4 y survey (from 1997 to 2000) of coral recruitment on Kenting reefs, Pocilloporidae and Acroporidae contributed >95% of the recruits [56]. Recent studies published in the region still lack the identification of coral recruits below the family level [57], [58], simply assuming that recruits were from the dominant genera. With the help of molecular tools, we were able to accurately identify six genera in four scleractinian families. Our identifications of *Montipora, Isopora,* and *Pavona* recruits are the first reliable records of these genera on settlement plates around Taiwan. If present in our study, we should also have been able to identify *Seriatopora* and *Stylophora* recruits, as COI sequences have recently shown their efficiency in delineating both genera [32].

While the use of COI would probably assist in the identification of several other genera in recruitement studies, the development of other molecular markers to identify recruits at the species level has only been applied to a minority of scleractinian lineages. Here, the use of PaxC for Acroporidae and ORF for Pocilloporidae demonstrates the potential utility of these new markers in delineating the species of *Isopora* and *Pocillopora*. PaxC has been previously used to delineate *Acropora* from *Isopora* by using *Isopora cuneata* as an outgroup [50]. Here, we were able to further separate recruits from both *Isopora cuneata* and *Isopora palifera* with a 100% bootstrap value. Schmidt-Roach et al. [53] recently used ORF sequences to delineate five distinct lineages within the ecomorphs of *Pocillopora damicornis* (see [59] for variation in ecomorph types). These lineages correspond to the reproductive behavior of *Pocillopora* on the Great Barrier Reef and in Western Australia [60]. Combined with their distinct microscopic skeletal structures, these different ORF types were subsequently described as different *Pocillopora* species [61]. Four of the five ORF types and a cluster *Pocillopora eydouxi*/*meandrina* have been identified in our study, revealing a higher diversity than previously thought in the Pocilloporidae recruits observed in coral communities around Taiwan [56]. Recruits of this family have typically dominated the reefs around Kenting, and our observations indicate that it seems to correspond mainly to a dominance of *Pocillopora* recruits (Fig. 7B, Table 3). This genus is composed of ‘weedy’ corals [62], characterized by species that brood their larvae over a prolonged period of time (November to March) in Kenting [46]. The status of Kenting reefs (see [40]) may be one of the reasons for the dominance of these opportunistic species in the pool of recruits as determined in this and previous studies [56], [71].

### Technical improvement

Although only 9.7% (24/248) of coral spats remained unidentified after DNA extraction, PCR amplification, and sequencing, we believe this value can be reduced further by improving the molecular analysis process. Because DNA extraction requires good quality DNA for accurate analyses, improving coral spat preservation after field collection and adapting the DNA extraction protocol to these tiny and degraded organisms could result in better yields. Our use of a high-salt extraction technique may not have been the best method for this biological material. Alternative DNA extraction protocols (such as the DNA extraction kit, organic extraction, and the whole genome amplification kit (REPLI-g, Qiagen, [39]) could result in better DNA quality/quantity and success in amplification and sequencing.

### Recruitment among sites

Low recruitment at OL was probably due to the low fecundity or low survivorship under the influence of warm water discharges from a nuclear power plant (Fig. 1), which has been shown to profoundly affect its benthic community [41], [74]. The thermal pollution may have contributed to the site's low recruitment rate, which is indicated by the high density of recruits observed at LDS (located adjacent to OL). LDS could also benefit from cyclonic eddies, which have been hypothesized to concentrate planula on the west side of the bay [63]. Alternativly, grazing by herbivores constitutes an alternative hypothesis for explaining low recruitment values, and is a well-known mechanism for accidentally reducing the number of coral recruits in the field [64]–[66]. Grazing marks from parrotfish and echinoids were readily observable on some of the plates in our study at OL and HBH. Therefore, the high coral cover observed at OL and HBH (Table 3) may have contributed to increased populations of herbivores, thus intensifying grazing pressure on coral recruits. Nevertheless, human-induced disturbance is also likely to play a major role in the observed patterns of recruitment. Nanwan Bay is exposed to a variety of anthropogenic impacts induced by coastal development, including water pollution and sedimentation, which are known to have already caused a shift away from corals at many sites around the bay [42], [45]. However, increased sampling and comparison of variances in recruitment with robust statistical analyses will be applied for better understanding whether the significant recruitment differences exist among sites.

By combining the use of existing tools, this report provides an original approach for precisely measuring effective coral recruitment on reefs. Further research based on this work may consider post-settlement mortality, which is usually disregarded in most studies on coral recruitment. In the future, finding additional molecular markers that are better suited for the identification of coral spat to genus and species is expected to compensate for historically lower levels of taxonomic resolution in previous research on early coral life stages and allow a more precise understanding of their role in the resilience of coral reef communities.
